# Immunomodulatory Effects by Photodynamic Treatment of Glioblastoma Cells In Vitro

**DOI:** 10.3390/molecules27113384

**Published:** 2022-05-24

**Authors:** Friederike Rothe, Ina Patties, Rolf-Dieter Kortmann, Annegret Glasow

**Affiliations:** Department of Radiation Oncology, University of Leipzig, 04103 Leipzig, Germany; friederike.rothe@medizin.uni-leipzig.de (F.R.); ina.patties@medizin.uni-leipzig.de (I.P.); rolf-dieter.kortmann@medizin.uni-leipzig.de (R.-D.K.)

**Keywords:** glioblastoma, ionizing radiation, IR, photodynamic, photosensitizer, vaccination, dendritic cells, tetrahydroporphyrin-tetratosylat, THPTS, GL261, PD-L1, PD-1

## Abstract

Multimodal treatment adding immunotherapy and photodynamic treatment (PDT) to standard therapy might improve the devastating therapeutic outcome of glioblastoma multiforme patients. As a first step, we provide investigations to optimize dendritic cell (DC) vaccination by using PDT and ionizing radiation (IR) to achieve maximal synergistic effects. In vitro experiments were conducted on murine glioblastoma GL261 cells, primary DCs differentiated from bone marrow and T cells, isolated from the spleen. Induction of cell death, reactive oxygen species, and inhibition of proliferation by tetrahydroporphyrin-tetratosylat (THPTS)-PDT and IR were confirmed by WST-1, LDH, ROS, and BrdU assay. Tumor cargo (lysate or cells) for DC load was treated with different combinations of THPTS-PDT, freeze/thaw cycles, and IR and immunogenicity analyzed by induction of T-cell activation. Cellular markers (CD11c, 83, 86, 40, 44, 69, 3, 4, 8, PD-L1) were quantified by flow cytometry. Cytotoxic T-cell response was evaluated by calcein AM assay. Immunogenicity of THPTS-PDT-treated GL261 cells lysate was superior to IR-treated lysate, or treated whole cells proven by increased DC phagocytosis, T-cell adhesion, proliferation, cytolytic activity, and cytokine release. These data strongly support the application of PDT together with IR for optimal immunogenic cell death induction in tumor cell lysate used to pulse DC vaccines.

## 1. Introduction

Glioblastoma multiforme (GBM) is the most aggressive (WHO grade IV) and most common malignant primary brain tumor in adults with an incidence rate of 3.22 per 100,000 people in the United States [1]. Despite intensive standard therapy consisting of surgical resection, followed by ionizing radiation (IR) with concomitant and adjuvant temozolomide (TMZ) therapy [2,3], it remains incurable. Depending on MGMT-promotor methylation, median survival rates of only 14 to 48 months are reached [4,5]. GBM cells are highly radio-resistant and invade the surrounding brain tissue, resulting in high numbers of recurrences occurring in 85% within the resection margin [6,7]. New therapeutic approaches to improve the long-term survival of patients, especially to reduce local relapses, are urgently necessary. Consequently, we investigate here a novel multimodal strategy, which may extend the standard therapy by photodynamic therapy (PDT) and dendritic cell (DC) vaccination. Various studies have demonstrated the activation of specific antitumor immunity by autologous DC vaccination in several rodent models [8,9,10]. DC vaccines mostly use monocyte-derived dendritic cells pulsed with RNA, selected cancer-associated antigen peptides, or with whole tumor lysate, the latter irradiated or freeze/thaw-treated to induce cell death [11]. The efficacy of DC vaccination largely depends on the immunogenicity of the DCs and therefore of the antigen preparation they are pulsed with [9,10,12]. Clinical studies showing a median overall survival (OS) of 22% in patients with relapsed GBM [13] and of 46.2% in newly diagnosed GBM [14] additionally treated with DC vaccines underline the need for multimodal first-line treatment approaches to induce long-term tumor control [15].

PDT uses a topically or systemically administered nontoxic photosensitizer (PS) which is activated by visible light of a specific wavelength and preferably accumulates in tumor cells. In the presence of oxygen, activation of the PS leads to formation of reactive oxygen species (ROS), causing cytotoxic cell death, damage of tumor vascularization, and an inflammatory reaction [16,17,18]. Furthermore, immunogenic cell death (ICD) triggered by PDT releases damage-associated molecular patterns (DAMPs), that activate innate and adaptive immunity [19,20]. So far, photosensitizers, such as 5-ALA, are mainly established in the treatment of GBM as part of fluorescence-guided surgery (FGS) to reach maximal tumor resection [21]. Nevertheless, interstitial PDT in local GBM recurrences [22], as well as intraoperative PDT, using a fiber attached with a laser diffusion balloon or through a lens into the cavity after brain tumor resection, is technically feasible [23,24]. The beneficial effect of traditional photosensitizers (e.g., protoporphyrin IX, talaporfin, photofrin) for PDT of GBM, however, is very limited, mainly because the commonly applied light wavelengths of 630–690 nm reach a tissue penetration of only up to 5 mm. PSs with maximal absorbance at higher wavelengths may increase the penetration depth. Moreover, alternative excitation sources such as chemo-/bioluminescence [25], isotopes emitting Cherenkov radiation [26] or X-rays, which are transduced into visible photons by scintillating nanoparticles, are under development [27]. Tetrahydroporphyrin-tetratosylat (THPTS) is a water-soluble, positively charged, near infra-red PS with a strong absorbance band at 760 nm, allowing tissue penetration of up to 13 mm [28,29]. THPTS showed preferential accumulation in tumor cells of C26 colon carcinoma versus normal tissue, especially in mitochondria [30,31]. In vitro and in vivo studies already demonstrated the efficacy of THPTS-PDT, for example in a rat bladder cancer model and in human retinoblastoma cells [32,33,34]. Concerning GBM, in vitro studies proved a significant reduction of tumor cell clonogenicity by THPTS-PDT combined with IR, compared to single treatments. THPTS-PDT decreased proliferation, metabolic activity, and clonogenic survival. Cell death was induced by apoptosis and autophagy and could be confirmed in vivo in a C6 GBM rat model [29]. Due to these properties, application of THPTS for PDT and in combination with a DC vaccination could extend the standard therapy. Thereby, surgical resection, IR, and chemotherapy (TMZ) would reduce the tumor volume and inhibit the proliferation of tumor cells. Local THPTS-PDT after surgery could eradicate the surviving and infiltrating GBM cells, adjacent to the resection cave. THPTS-PDT-induced activation of the immune system might then synergize with the DC vaccination, inducing long-term anti-tumor response and prevention from relapses.

As a first step to generate an effective DC vaccine, it was our aim to optimize the activation of immune cells by pre-treatment of tumor cells with THPTS-PDT and/or IR. Therefore, we investigated the phagocytotic activity, DC maturation, T-cell activation, and cytotoxic response after activation of murine DCs with differentially pre-treated murine Gl261 glioblastoma cell lysate or cells in vitro. Subsequently, these results could serve as the basis for further investigations combining these improved DC vaccines with IR and PDT in vivo in a mouse model.

## 2. Results

### 2.1. Dose Dependent Effects of THPTS-PDT and IR on GL261 Cells

**Figure 1:**
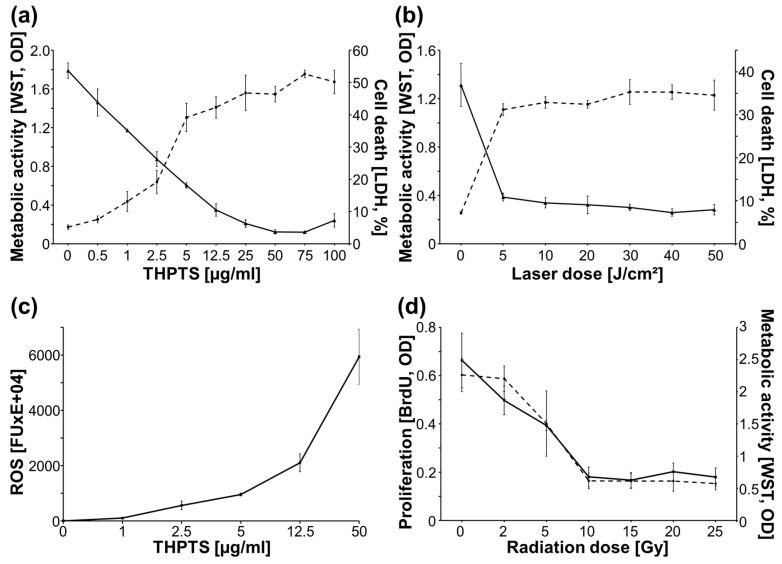
Effect of THPTS-PDT and IR on GL261 cells. THPTS (**a**) and laser (**b**) dose-dependent decrease of metabolic activity, increase of cell death (LDH, dashed line); and (**c**) increased release of reactive oxygen species (ROS) after THPTS-PDT; (**d**) inhibition of proliferation and metabolic activity (WST-1, dashed line) by irradiation; mean ± SEM of one experiment in triplicates.

To evaluate the lethal dose combination of THPTS and laser light in GL261 cells, the dose dependency of THPTS-PDT was analyzed by metabolic activity (WST-1 assay) and cell death induction (LDH-assay). THPTS concentrations from 0 to 100 µg/mL were applied, followed by a laser light dose of 30 J/cm². Maximal cell death induction was achieved at a THPTS concentration of 50 µg/mL, Figure 1a. Using light doses from 0 to 50 J/cm^2^ after THPTS treatment (25 µg/mL), a lethal laser light dose of 20 J/cm^2^ (760 nm) was determined, Figure 1b. Thereby, THPTS-PDT (0–50 µg/mL) induced a dose-dependent release of reactive oxygen species (DCFH-DA assay), Figure 1c. Next, we evaluated the effect of IR on cell proliferation (BrdU assay) and on cell viability (WST-1 assay), using IR doses from 0 to 25 Gy. A maximum inhibition of cell proliferation and decrease of metabolic activity/viability was achieved at 10 Gy, Figure 1d.

Based on these results, GL261 lysate generation was realized, using THPTS concentrations of 50 µg/mL, laser doses of 20 J/cm^2^, and IR doses of 15 Gy ensuring complete killing of tumor cells used for DC pulsing.

### 2.2. T-Cell Generation, Differentiation and Maturation of DCs

The purity of T cells isolated from murine spleen by magnetic cell separation was >85% (87.15 ± 1.20% CD3-positive cells). The proportion of CD4- and CD8-positive cells was 56.5 ± 9.3% and 33 ± 7.1%, respectively (mean ± SED, *n* = 2). The expression of PD-1 on freshly isolated CD3-positive cells was 10%.

**Figure 2:**
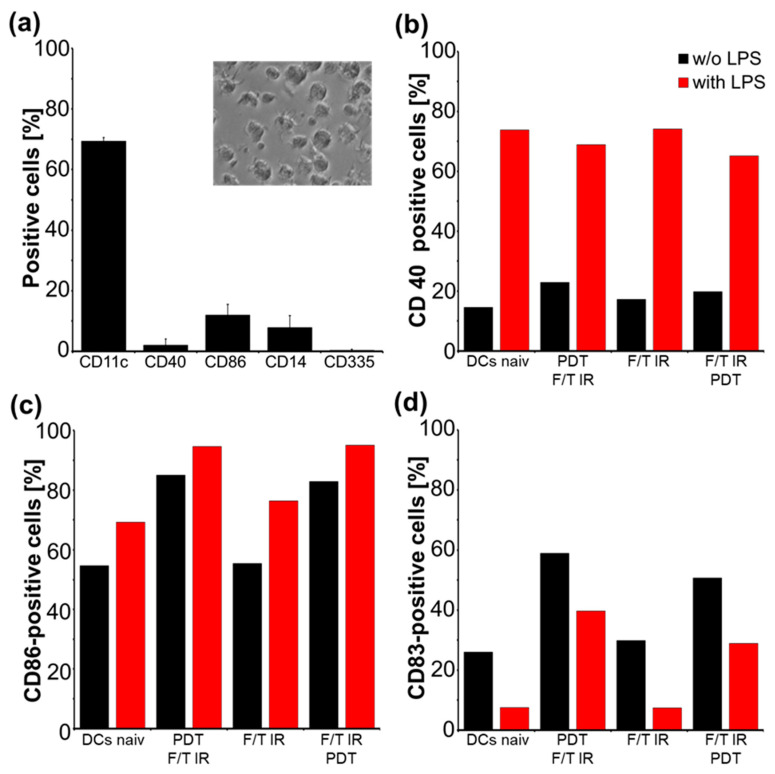
Differentiation and maturation of DC. (**a**) Quantification of DC (CD11c), monocytes (CD14), natural killer cells (NK, CD335) and number of CD40-, CD86-positive, activated DCs, *n* = 3; phase contrast image of differentiated DCs, original magnification: 40×; (**b**) Effect of GL261 lysate, cell antigen preparation and LPS activation on CD40; (**c**) on CD86; and (**d**) on CD83 expression in CD11c-positive DCs, flow cytometry (FC) analysis, one representative experiment of two.

The quality of DC differentiation was confirmed by analysis of differentiation markers 7 days after isolation of bone marrow cells and subsequent cultivation with 200 U/mL murine GM-CSF (standard protocol), Figure 2a, mean ± SEM, *n* = 3. DC surface marker CD11c was expressed on 69.3 ± 1.2%, activation markers CD40 (2.03%) and CD86 (11.93%) were on 2.03% and on 11.9% of generated cells. Only 7.8 ± 4% expressed monocyte surface marker CD14. Induction of natural killer (NK) cells was minimal (0.35% CD335-positive cells). Differentiated DCs show stellate morphology with dendrites ready for antigen uptake. DC activation and maturation were analyzed 24 h after pulsing DCs with differentially treated GL261 lysates, with/without LPS activation. Pulsing with PDT-treated GL261 lysates increased the expression of CD40, CD86, and CD83 on DCs (CD11c-positive cells) compared to only IR-treated lysates and naïve DCs. Addition of LPS (50 ng/mL) massively enhanced CD40 and slightly CD86 levels, whereas CD83 levels were reduced, Figure 2b–d.

### 2.3. Phagocytic Activity of DCs

**Figure 3:**
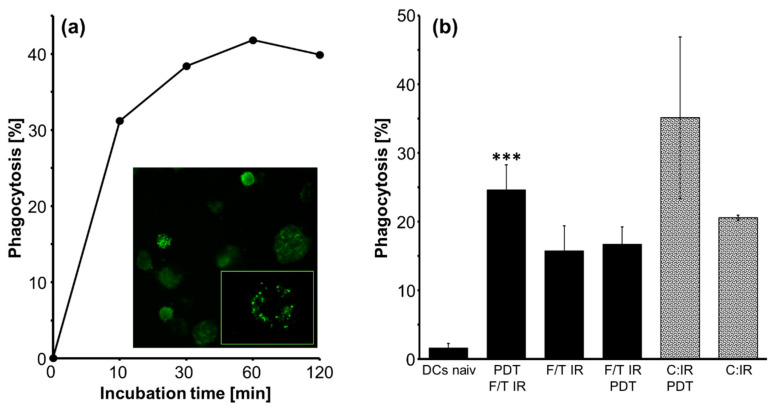
Phagocytosis. Quantification of phagocytic active (CFSE-positive) DCs/total number of DCs (100%). (**a**) Time-dependent phagocytosis of DCs shown in one representative experiment of two. CFDA-positive vesicles (green) prove uptake of lysate by DCs, image, original magnification: 40×, insert: 100×; (**b**) Quantification of phagocytic active (CFSE-positive) DCs/total number of DCs, mean ± SEM, *n* = 4; ***, *p* ≤ 0.001 compared to F/T IR.

To evaluate the influence of GL261 lysate preparation on the phagocytic activity, DCs were incubated with differentially treated and CFDA-SE-labelled GL261 lysates/cells, Figure 3.

We investigated the optimal incubation time for phagocytosis by incubating differentiated DCs with labelled GL261 lysates for 10, 30, 60, and 120 min at 37 °C. Evaluation by flow cytometry (FC) indicated that incubation times longer than 60 min (41.8% CFDA-SE-positive cells of total cell number) did not further enhance the lysate uptake by the DCs. Prolongation of incubation time up to 6 h even resulted in the death of 90% of the DCs during maturation. Therefore, subsequent DC pulse experiments were performed with an incubation time of 60 min, Figure 3a. CFSE-labeled phagocytosis vesicles in DCs confirmed the uptake of lysate, fluorescence image (original magnification: ×40, insert: ×100), Figure 3a. Analysis of different lysate protein concentrations using 50,000 DC/50 µL and 0 to 200 µg GL261 lysate (F/T, IR) showed a maximum specific uptake at a protein concentration of 1 mg/mL, which was therefore applied for all phagocytic experiments.

Lysate or cell-pulsed DCs showed a significant increase of CFDA-SE uptake compared to naïve DCs in FC analysis (*p* ≤ 0.01, *n* = 4). The highest phagocytic activity with 24.6 ± 3.67% (mean ± SEM) CFSE-positive cells was detected in DCs pulsed with THPTS-PDT-treated lysate prior to F/T cycles and IR treatment. It was significantly higher compared to IR lysate treatment (15.7 ± 3.6; *p* ≤ 0.001, *n* = 4). In addition, pulsing with tumor cells resulted in slightly higher amounts of phagocytic activity in DCs pulsed with IR/PDT-treated (35.1 ± 11.8%) compared to IR-treated cells (20.6 ± 0.4, *n* = 2), although not reaching significance, Figure 3b.

We observed a downregulation of the DC marker CD11c by fluorescence microscopy, 3 h after activation/induction of DC maturation by GL261 lysate uptake. FC analysis revealed a lower mean fluorescence intensity (MFI) in GL261 lysate (treatment: PDT F/T, IR)-pulsed DCs (MFI = 35,336), compared to the naïve DC control (MFI = 115,691), indicating a decreased level of CD11c expression and even a loss of CD11c in one fifth of lysate-pulsed DCs.

### 2.4. DC/T-Cell Priming: Activation and Proliferation of T Cells

**Figure 4:**
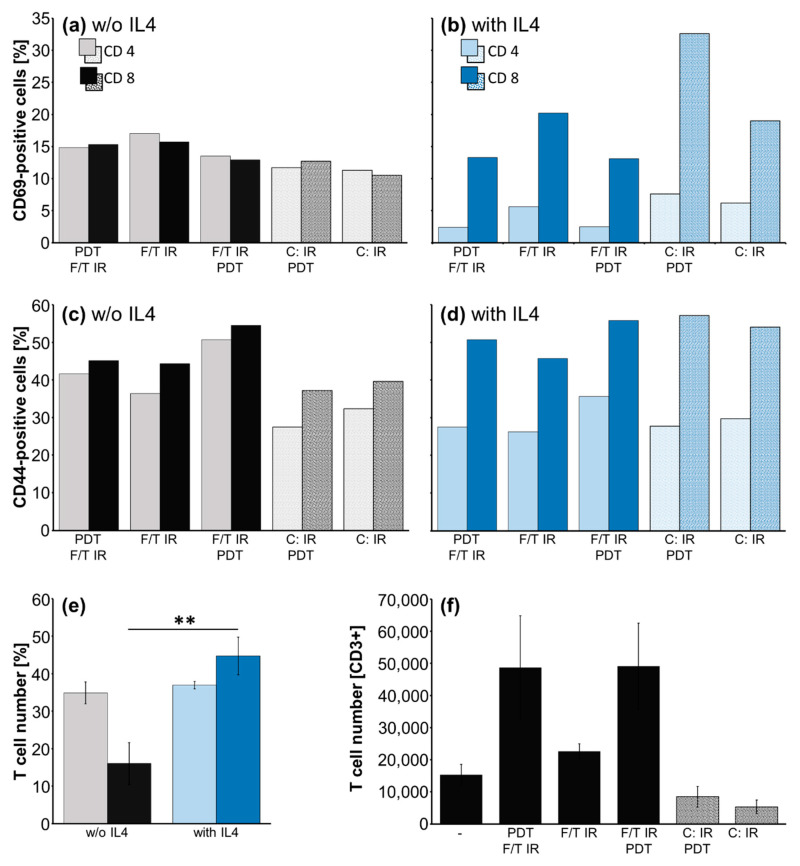
Induction of T-cell activation markers and proliferation. (**a**–**d**) Effect of lysate and cell antigen preparation and addition of IL4 on CD69 (**a**,**c**) and CD44 (**b**,**d**) T-cell activation markers in CD4- and in CD8-positive T cells of one representative experiment; (**e**) Effect of IL4 on CD4 and CD8 T-cell numbers, mean of five preparation groups ± SEM, **, *p* ≤ 0.01; (**f**) T-cell number after priming with DCs pulsed with indicated antigen preparations including LPS, or (-) LPS only, mean ± SEM, *n* = 2 (**e**,**f**).

The expression activation markers CD44 and CD69 in CD4- and CD8-positive T cells 7 days after priming with tumor lysate or cell-pulsed DCs (including LPS 50 ng/mL) is shown in Figure 4. We did not found differences in the T-cell activation or CD4/CD8 ratio depending on the tumor cell/lysates treatments. The addition of IL4 (200 ng/mL) during priming did not enhance the CD69 and CD44 expression in CD8 and decreased it in CD4 cells (mean over all treatment groups, *p* ≤ 0.05). The differential lysate or cell antigen treatments for DC pulsing had no effect on both markers, Figure 4a–d. Supplementation of LPS during maturation and priming (50 ng/mL) enhanced the expression of CD44 by 1.6/2.1-fold (CD4/CD8) and of CD69 2.9/5.3-fold (CD4/CD8).

The ratio of CD4/CD8 remained largely unchanged in different antigen treatments (lysate CD4/CD8: 2.3; cells CD4/CD8: 1.9) versus priming with naive DCs (2.1) and after repriming but strongly decreased after IL4 supplementation, naïve DCs+IL4 (0.4). Administration of IL4 to T-cell priming resulted in an expansion of CD8-positive T cells from 16.02% to 44.7% whereas the CD4 cell population remained unchanged (34.9 without, 36.9% with IL4), CD4/CD8 ratio one experiment with five treatment groups, Figure 4e.

Analysis of the T-cell number after priming T cells with lysates and tumor cell-pulsed DCs reveals induction of T-cell proliferation by PDT treatment of GL261 lysates independent if applied before or after F/T cycles, Figure 4f, mean ± SEM, *n* = 2.

To investigate if our treatments induced suppressive T-cell responses, we measured the number of regulatory T cells (Tregs) after priming with pulsed DCs. The proportion of CD152 (Tregs) in the CD3-positive cell population (T cells) was minimal after first priming (5.24 ± 0.02%) and not enhanced compared to naive DCs (6.08 ± 0.04%). In addition, a second T-cell priming did not increase the proportion significantly (5.53 ± 0.02% in treated DCs versus 8.03 ± 0.03% in naive DCs (mean ± SEM, *n* = 2).

### 2.5. Cytokine Release

**Figure 5:**
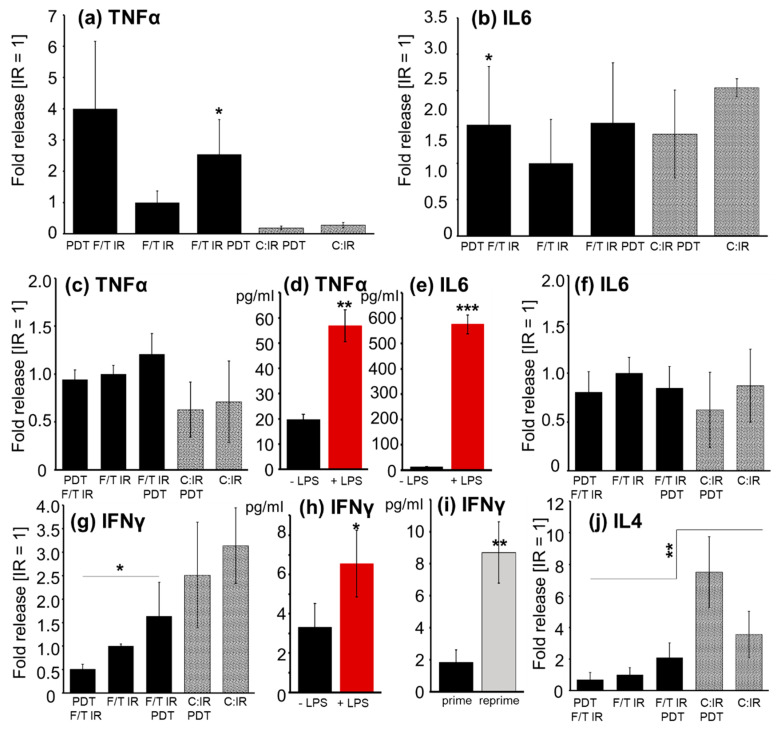
Cytokine release. Fold release represents relative values (IR = 1) ± SEM; otherwise mean ± SEM is given, *n* ≥ 3.TNFα (**a**) and IL6 (**b**) release of dendritic cells, 24 h after pulsing with different antigen preparations (PDT,F/T,IR, F/T,IR; F/T,IR, PDT): GL261 lysate (black bars); GL261 cells (dotted bars); *, *p* ≤ 0.05 relative to F/T IR treatment. TNFα (**c**,**d**) and IL6 (**e**,**f**) release by DC/T cells 24–72 h after priming of T cells with DCs; (**c**,**f**) Effect of antigen preparation; (**d**,**e**) joint analysis of LPS effect in all five antigen preparations of one experiment; **, *p* ≤ 0.01; ***, *p* ≤ 0.001. IFNγ (**g**,**h**,**i**) and IL4 (**j**) release by 72 h after T-cell repriming: Effect of antigen preparation on IFNγ (**g**) and IL4 (**j**) release; (**h**) IFNγ release after LPS administration compared to without; *, *p* ≤ 0.05; and (**i**) after reprime compared to single prime; **, *p* ≤ 0.01.

DCs, 24 h after pulsing, Figure 5a,b: Pulsing of DCs with PDT/IR-treated lysate enhanced significantly the IL6 and TNFα release compared to IR-treated samples after 24 h, Figure 5a,b (*n* = 3, *p* ≤ 0.05; IL6: mean IR = 4369 pg/mL; TNFα: mean IR = 968 pg/mL).

Dendritic and T cells, 24–72 h after priming, Figure 5c–f and 72 h after repriming, Figure 5g–j.: Functional integrity of T cells was demonstrated by their stimulatory potential towards LPS and towards repetitive antigen-loaded DC priming (repriming), Figure 5d,e,h,i. Thereby, LPS increased TNFα (*p* < 0.01) and IL6 (*p* < 0.001) release in single primed T cells (Figure 5c,d), whereas antigen preparation had no significant effect Figure 5c,f.

In reprimed T cells, PDT did not significantly alter the IFNγ release versus IR treatment alone, however, PDT after the freeze/thaw cycles (IR F/T PDT) enhanced IFNγ levels significantly compared to PDT before F/T (PDT F/T IR), *n* = 3, *p* ≤ 0.05, Figure 5g. Pulsing of DCs with cellular antigen resulted in 1.9-fold higher IFNγ release than corresponding pulsing with GL261 lysate (5.0 ± 1.8 pg/mL vs. 2.67 ± 0.63 pg/mL, black bars vs. dotted bars, *n* = 3, *p* ≤ 0.01, Figure 5g). LPS, added to DCs during pulsing led to a 2-fold enhancement of IFNγ release by T cells, *p* ≤ 0.05, *n* = 3, Figure 5h. Restimulation (repriming) of T cells by antigen-loaded DCs, increased the IFNγ levels by 4.7-fold, compared to single priming, *p* ≤ 0.01, *n* = 3, Figure 5i.

IL4 values were low and, after T-cell priming, it took them 5 d to reach detection limit. Pulsing of DCs with cellular antigen resulted in 5.2-fold higher IL4 release than corresponding pulsing with GL261 lysate (17.4 ± 3.9 pg/mL vs. 3.4 ± 1.8 pg/mL, *n* = 3, *p* ≤ 0.01) Figure 5j.

### 2.6. Cytotoxic T-Cell Activity

**Figure 6:**
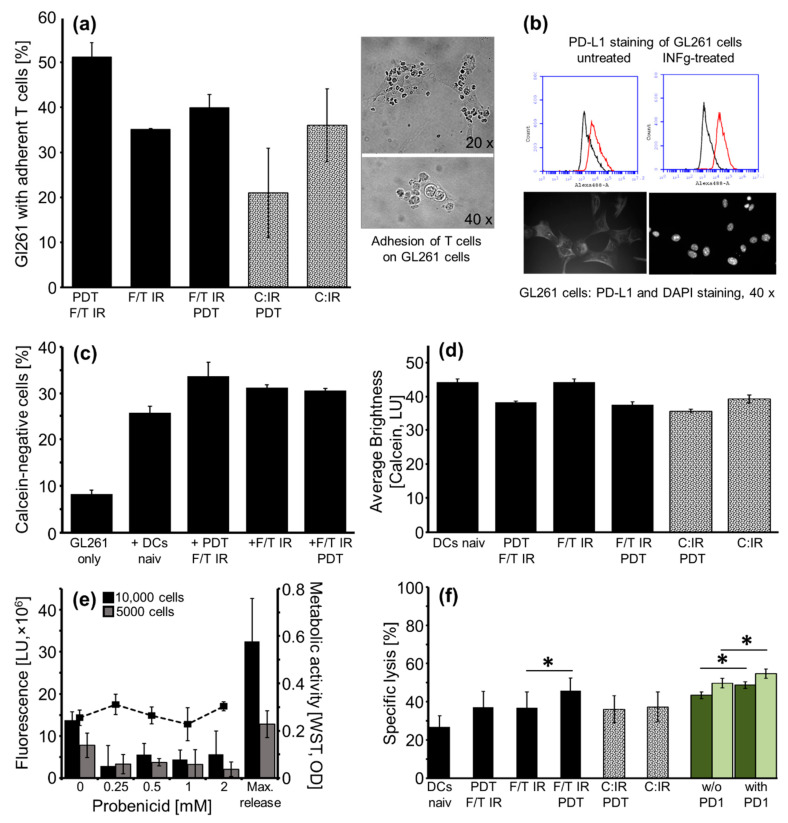
Cytotoxic T-cell activity. (**a**) T-cell adhesion on GL261 cells depending on priming with differentially pulsed DCs, mean ± SEM, triplicate experiment, representative of two; phase contrast photography of adherent T cells on GL261 cells, original magnification 20× and 40×; (**b**) Expression of PD-L1 (red) on GL261 cells without and with IFNγ stimulation and corresponding IgG-negative control (black), below image of PD-L1 and corresponding DAPI fluorescence stained GL261 cells; (**c**) Flow cytometric analysis of calcein loss (**c**) and quantification of calcein retention by fluorescence microscopy (**d**) in GL261 cells, 4 h after incubation with primed T cells; (**e**) Dose-dependent effect of probenecid on background fluorescence, caused by spontaneous calcein leakage, in the supernatant of calcein-labelled GL261 cells; dashed line, effect on metabolic activity; (**f**) Specific cytolytic activity of primed T cells dependent on antigen preparation for DC pulsing, *n* = 5; joint analysis of 4 h (dark green) and 24 h (light green) lysis with and without PD-1 block over all five treatment groups, mean ± SEM, *, *p* ≤ 0.05.

Cytotoxic T-cell activity starts with the attachment of T cells on Gl261 cells. The proportion of adherent T cells in the GL261 tumor cells, depending on priming with differentially pulsed DCs, is shown in Figure 6a together with a phase contrast photograph of the fixed Gl261 cells with adherent T cells. Overall, the T-cell adherence was stronger using lysates than tumor cells for DC loading. T cells primed with DCs that have been pulsed with additionally PDT-treated lysates showed the greatest adherence to GL261 tumor cells: PDT; F/T; IR with 51.2 ± 3.1% followed by F/T; IR; PDT with 40.0 ± 2.9% (mean ± SEM, experiment in triplicates).

PD-L1 is an immune-inhibitory receptor ligand, which is expressed on many types of cancer cells, also on GBM and has been shown to block the anti-tumor activity of immune cells by binding to PD-1. FC analysis revealed 23.2% PD-L1-positive GL261 cells and the addition of IFNγ (50 ng/mL) for 24 h more than doubled the amount to 53% PD-L1-positive cells. Upregulation of PD-L1 by IFNγ was confirmed by immunofluorescence microscopy, Figure 6b.

Cytotoxic T-cell response was quantified in GL261 cells by a loss of fluorescence using FC, Figure 6c, by the retention of calcein AM measuring average brightness using fluorescence microscopy, Figure 6d, and by the calcein concentration in the supernatant, Figure 6f. Thereby, the strongest reduction of intracellular calcein concentration, indicating substantial cell lysis, was consistently demonstrated after incubation with T cells primed with PDT IR-treated lysate, compared to IR-only-treated lysate, Figure 6c,d.

To minimize spontaneous calcein release (calcein leakage), resulting in high background fluorescence in the cytotoxic T-cell lysis assay, we investigated the use of the trans-membranous channel/anion blocker, probenecid. Spontaneous release was reduced 4.8-fold/2.4-fold (10,000/5000 seeded GL261 cells) adding probenecid at a concentration of 0.25 mM. Thereby, the difference in maximum release versus spontaneous release increased from 2.4- to 11.4-fold/1.7- to 3.9-fold (10,000/5000 seeded GL261 cells). No toxicity of probenecid was found for incubation times of up to 4 h by WST-1 assay, Figure 6e.

Consequently, in the following experiments 10,000 GL261 cells were seeded 24 h prior to calcein-release assay and 0.25 mM probenecid was added right before calcein AM. Cytotoxic T-cell assay revealed significantly enhanced lysis by T cells primed with DCs pulsed with F/T IR PDT-treated lysate compared to F/T IR treatment, *n* = 5, *p* ≤ 0.05. All antigen preparations led to significantly higher lysis than naive (LPS only) DCs, *p* ≤ 0.05. Administration of PD-1 antibody blocking PD-1 binding and interaction with PD-L1 significantly reduced GL261 cell lysis by T cells. Tumor-specific lysis increased with T-cell coincubation time (4 and 24 h), Figure 6f.

## 3. Discussion

Multitargeted therapy approaches might be a successful strategy to improve the overall survival time for glioblastoma patients. To add a new pillar to the current therapy, we tested here the development of an effective antitumor vaccine, using the model of GL261 cells and corresponding murine DCs in vitro.

Photodynamic therapy is known to enhance anti-tumor immunity in vivo [35] and has been tested here, to exploit its immunogenic potential for improved DC vaccines. Of particular importance was the application of a photosensitizer with high therapeutic depth (THPTS), which, in an adjuvant residual tumor THPTS-PDT setting, might contribute to home the immune cells to the PDT-treated areas. The quality of tumor cell antigens is of special importance for the induction of an effective anti-tumor activity in DC vaccines [10,12]. Consequently, we have herein optimized freeze/thaw, irradiation, and THPTS-PDT schedules, as well as the application of tumor whole cell lysate versus dead tumor cells.

### 3.1. THPTS-PDT and Irradiation in GL261 Cells

Here we could demonstrate that THPTS-PDT efficiently induced cell death in the murine glioblastoma GL261 cells, confirming our previous data in three different human glioblastoma cell lines [29]. Metabolic, proliferation, and LDH-release assays approved complete cell killing at the applied conditions, Figure 1. THPTS-PDT leads to apoptotic and necrotic cell death, and synergizes with irradiation [29]. For the generation of a dendritic cell vaccine, the residual surviving, tumor cells were additionally treated with irradiation and/or underwent freeze/thaw cycles before pulsing DCs, complying with clinical protocols [36].

### 3.2. Dendritic Cell Response after Pulsing

DC differentiation and maturation was confirmed by the expression and LPS-induced response of corresponding surface markers (CD40, CD86) and cytokine release [37], Figure 2 and Figure 5. The enhanced activation markers, CD83 and CD86, found in DCs pulsed with PDT F/T IR- versus IR-only-treated lysates, Figure 2, resemble findings in 5-ALA/PDT-treated glioma spheroids, although activation there was not compared to the irradiated samples [38].

In accordance, phagocytosis of DCs was also significantly enhanced by PDT F/T IR-treated compared to IR-treated lysate, Figure 3. This immunogenic functional stimulation is in line with recent findings after hypericin-based PDT and has been partly attributed to the induction of damage-associated patterns (DAMPs) [12]. Induction of immunogenic cell death (ICD) seems to be a common feature of PDT, although with varying efficiency [9,12,16], possibly depending on the subcellular localization of the PS [20]. It has herein also been confirmed for THPTS-PDT by ROS species induction and LDH release, Figure 1, which is in line with previous data showing that THPTS-PDT enhanced HSP70 expression in retinoblastoma cells [34].

Enhanced proinflammatory cytokine release 24 h after pulsing DCs underlined the immunogenic potential of PDT-treated lysates, compared to IR treatment, Figure 5a,b. In contrast to phagocytosis, the time point of PDT (before or after F/T) was not relevant here.

The detected partial loss of CD11c after DC activation by lysate might serve as a further marker of DC activation. CD11c has been reported to be downregulated in response to TLR agonists, such as LPS and tumor-derived DAMP proteins [39,40].

### 3.3. T-Cell Response after Priming

LPS is widely applied as a coactivator in DC vaccines and, as expected, induced CD40 expression on the DCs here, which is known to activate the Th1 CD4-positive cells to trigger subsequent cytotoxic (CD8-positive) T-cell expansion [41].

Activated DCs led to adequate T-cell response, resulting in enhanced T-cell numbers in PDT-treated lysate-pulsed DCs, Figure 4f. Control stimulation by LPS significantly enhanced CD44 and CD69 T-cell activation markers on CD4- and CD8-positive T cells, as well as Il6 and TNFα release, confirming robust DC/T-cell response.

The IL4-induced activation of CD8-positive T cells and reduction of CD4/CD8 ratio (Figure 4) was not followed up, because it did not translate into enhanced cytotoxic T-cell response. IL4 had already been suggested to increase the stimulatory potential of DCs, although mainly under serum-free conditions [42]. Generally, IL4 is considered to trigger the Th2 response [43] but it has also been described to be a potent stimulator of CD8 cell proliferation in vivo [44] which again is attributed to its action on DC maturation [45].

PDT lysate-pulsed DCs produced slightly more Th1 CD4-positive cell-specific cytokines (TNFα, IFNγ), whereas whole cell-pulsed DCs induced stronger IL6 and IL4, indicative of a Th2 response. This implies that the lysate is the better antigen for DC pulsing to achieve an anti-tumor-specific, cytotoxic (CD8-positive) T-cell response

Repriming of T cells with antigen-loaded DCs or priming with LPS enhanced the IFNγ release (Figure 5), specific for Th1 CD4-positive T-cell activation, expected to subsequently enhance cytotoxic CD8-positive T-cell expansion. Nevertheless, the CD4/CD8 ratio remained largely unchanged, possibly requiring longer periods and repetitive stimuli. The IL4 release was significantly higher in DCs pulsed with tumor cells than with lysate, indicating a Th2 response going along with the slightly lower cytotoxic activity of T cells pulsed with tumor cells than with lysate (see Section 3.4).

### 3.4. Cytotoxic T-Cell Response

Immunogenic cell death (ICD) eliciting danger signals has been suggested to play a crucial role for the improvement of dendritic cell vaccines and effective anti-tumor T-cell responses [9]. PDT is increasingly recognized as a main inducer of ICD [20,24].

Accordingly, our results of the cytotoxic T-cell response could confirm an enhanced cytotoxic potential of T cells primed with F/T IR PDT-treated lysate-pulsed DCs compared to IR-treated lysate, Figure 6. Results from Vandenberk et al. showed that higher immune responses depend on IR treatment-induced protein carbonylation, a feature of oxidation-associated molecular patterns (OAMPS), which are partly removed by repetitive F/T cycles [10]. Therefore, we applied IR after the F/T cycles, when preparing tumor lysate for DC activation. The time point of PDT treatment (before or after the F/T cycles), however, was of minor importance. Whilst the DC phagocytosis was more strongly stimulated by the PDT F/T IR lysate, the T-cell activation regarding cytokines was independent and, regarding cytotoxic lysis, slightly stronger when using F/T IR PDT lysate.

Additionally, we compared PDT-treated tumor cells and tumor cell lysate for DC pulsing and subsequent T-cell priming. We hypothesized that the presentation of the tumor cell surface to DCs would induce a more tumor-specific cytolytic T-cell response, compared to presentation of the whole cell lysate. Our experiments did not confirm this hypothesis. Presumably, the intracellular components released by using tumor cell lysate led to a high costimulatory effect resulting in the stronger T-cell activation demonstrated. Therefore, the administration of (PDT/IR-treated) tumor cell lysate to pulse DCs seems to be an effective way for stimulation of the cytotoxic T-cell response and is in fact much easier to realize in the clinical setting.

Confirming findings by Qian et al., we could demonstrate PD-L1 protein expression on the surface of GL261 tumor cells and upregulation by IFNγ [46]. Even when PD-L1 expression is initially low (2.77% PD-L1 expressing cells in GBM, [47]), it can be upregulated by activated tumor-infiltrating lymphocytes [48].

The delivery of antibodies against PD-L1 to the brain through the blood–brain barrier might prove difficult. Instead, blocking of the PD-L1/PD1 axis targeting the immune cell site with inhibitory PD-1 antibody could be more advantageous. Accordingly, we found that administration of PD-1 inhibitory antibody to primed T cells during interaction with GL261 tumor cells enhanced significantly their cytolytic activity, Figure 6e. This relatively moderate effect might be pronounced in long-term in vivo conditions, when infiltrating T cells enhance PD-L1 expression on glioblastoma cells.

A multimodal approach employing surgery, IR, PDT of tumor bed, PDT/IR-treated lysate DC vaccine, and PD-1 inhibitor might offer additional synergistic responses. One example shown by others is the activation of macrophages by laser irradiation [49] as an additional benefit of such a multi-targeted strategy. Furthermore, novel concepts enhancing the intra-tumoral oxygen supply, might help to overcome hypoxia-induced tumor cell resistance to PDT and IR-treatment [18], especially if both methods are simultaneously applied in X-PDT [27].

In conclusion, we could demonstrate enhanced phagocytosis induction in DCs and cytotoxic T-cell activation by DCs pulsed with THPTS-PDT/IR compared to IR-only-treated tumor lysate. The immunogenicity of tumor lysate was further strengthened by the immune checkpoint blockade. Our results warrant in vivo verification, using a DC vaccine and THPTS-PDT/IR-treated GL261 lysate on a glioblastoma mouse model within a future project.

## 4. Materials and Methods

### 4.1. Animals

C57BL/J6 mouse breeding was performed in the animal facility of the Faculty of Medicine, University of Leipzig. Animals were housed in a 12 /12 h light/dark cycle with access to food and water ad libitum. The local authorities (Landesdirektion Sachsen T12/17) had approved all experiments in advance.

### 4.2. GL261 Cell Culture and Supplements

The murine GL261 glioma cell line was purchased from DSMZ, Braunschweig Germany (GL-261, ACC 802 and cultivated at 37 °C under 5% CO_2_ in DMEM (Lonza, Basel, Switzerland) supplemented with 10% heat-inactivated fetal calf serum (FCS; Sigma-Aldrich, St. Louis, MO, USA), 100 U/mL penicillin, 100 µg/mL streptomycin (Lonza) and 2 mM L-glutamine (Sigma-Aldrich).

### 4.3. THPTS-PDT

THPTS (C_72_H_70_N_8_O_12_S_4_; molecular weight 1367.77 Da; 5,10,15,20-Tetrakis(1-methyl-3-pyridyl)-21H,23H-7,8,17,18-tetrahydroporphyrin tetratosylat) was purchased from TetraPDT GmbH (Leipzig, Germany). Chemical structure and UV-VIS spectrum of THPTS are provided in Appendix A. For laser light application, a 2.5 W diode laser (LAMI, NTC laser division, obtained from TetraPDT GmbH, Rackwitz, Germany) at 760 ± 3 nm was used. After administration of THPTS, the cells were handled in the dark except for a filter-armed 600 nm emitting halogen spotlight to prevent excitation of THPTS, according to its spectra [31]. For THPTS-PDT, the cells were incubated with 50 µg/mL THPTS for 1 to 2 h in 15-mL tubes at 37 °C, if not otherwise specified. Before laser treatment, THPTS containing medium was renewed to avoid self-shielding and cells were transferred to 1.5-mL tubes. Light doses of 5–50 J/cm² were delivered at an intensity of <100 mW/cm^2^ (to avoid thermal effects), for up to 3 min.

### 4.4. Ionizing Radiation (IR)

For IR, a 150 kV orthovoltage X-ray machine (Xstrahl 200, Xstrahl, Ratingen Germany) was used. To irradiate GL261 tumor cells, a single dose of 15 Gy with a dose rate of 1.85 Gy/min was applied, if not otherwise indicated. Sham-IR was performed at equal conditions.

### 4.5. Metabolic Activity, Proliferation, Cell Death and Reactive Oxygen Species (ROS) Induction

The effect of THPTS-PDT on metabolic activity was quantified by WST-1 formazan assay (Roche, Basel, Switzerland). GL261 cells were seeded in 96-well plates (20,000 cells/well) and allowed to adhere for 24 h. THPTS-PDT (THPTS concentrations: 0–100 µg/mL; laser light doses: 0–100 J/cm^2^), 2 h drug light interval, was conducted. Then, 24 h later, WST-1 reagent was added (10 µL per 100 µL/well) and after 1 h of incubation absorbance was measured at 435 nm on a microplate reader (SpectraMax i3x, Molecular Devices, San Jose, CA, USA). In the case of IR treatment, 10,000 cells/well were seeded, IR doses of 0 to 25 Gy (dose rate 96-well plate: 1.5 Gy/min) were applied 24 h later and after 72 h, WST-1 assay was performed.

To analyze cell death induction by THPTS-PDT, LDH release assay (CytoTox-One Homogeneous Membrane Integrity Assay, Promega, Fitchburg, WI, USA) was used. The GL261 tumor cells were seeded and treated with THPTS-PDT, as described above. For maximum LDH release control, 2 µL of lysis solution were added per 100 µL/well and the plates were incubated for another 45 min at 37 °C, 5% CO_2_. Then, 50 µL of supernatant per well (100 µL original volume) was transferred to new opaque 96-well plates and 50 µL of CytoTox-One Reagent added. After incubation for 30 min at 37 °C, 5% CO_2_ fluorescence was recorded (Ex/Em = 560/590 nm) on a microplate reader (SpetraMax i3x).

Cell proliferation was measured by 5-bromo-2′-deoxyuridine (BrdU) proliferation assay (Roche). GL261 cells were seeded in 96-well plates (5000 cells/300 μL per well) and IR treatment (0–25 Gy) was conducted 24 h later. After 72 h, the medium was refreshed, BrdU labelling solution was added for further 24 h, and BrdU ELISA executed, according to the manufacturer’s instructions.

Induction of ROS by THPTS-PDT was detected by 2‘,7‘-dichlorofluorescin diacetate (DCFH-DA) assay (Sigma-Aldrich). The GL261 cells were seeded in 96-well plates (opaque, clear bottom; 5000 cells/150 μL per well). THPTS reagent (0 to 50 µg/mL) was added 48 h later and after 2 h of incubation, the medium was changed to serum-free DMEM without phenol red (200 µL/well) containing 10 µM DCFH-DA. Subsequently, laser treatment (10 J/cm^2^) was applied. Plates were incubated for 15 min at 37 °C, 5% CO_2_ and fluorescence was recorded (Ex/Em = 495/529 nm) on a microplate reader.

### 4.6. Preparation of GL261 Lysates

To generate lysates, GL261 cells were cultured in T175 flasks and harvested by flushing twice with PBS. Cell suspension was collected in a 50 mL tube, cells were counted and washed twice using PBS. The GL261 lysates were prepared in three different ways: (1) GL261 cells submitted to six consecutive freeze/thaw cycles (F/T: liquid nitrogen/37 °C) followed by 15 Gy IR (F/T IR); (2) cells subjected to THPTS-PDT prior to the F/T cycles and IR (PDT F/T IR); and (3) cells supplemented with THPTS before the F/T cycles and IR and exposed to laser treatment (PDT) afterwards (F/T IR PDT). THPTS treatment was applied to GL261 cells as described (THPTS-PDT) in 15-mL tubes in GL261 medium at a cell density of 10^7^ cells/mL. All other treatments (PDT IR F/T) were conducted in 1.5-mL tubes in serum-free DC culture medium. Afterwards, cell lysates were stored in aliquots, preventing rethaw/freeze, at −80 °C until use. Protein concentrations were determined by colorimetric Bradford protein assay (Sigma-Aldrich), corresponding to the manufacturer’s protocol.

### 4.7. GL261 Tumor Cell Treatment

GL261 cells (C) were seeded in 6-well plates (3 × 10^6^ cells/well) in GL261 culture medium and cells were exposed to 15 Gy (dose rate: 1.5 Gy/min) IR only (C:IR) or to IR followed by THPTS-PDT (C:IR PDT). Medium was changed to DC medium after IR. Experimental conditions were kept the same and the cells were handled in the dark.

#### CFDA-SE Labelling

Part of the cells was labelled with the fluorescenT-cell tracer CFDA-SE (Vybrant CFDA SE Cell Tracer Kit, Invitrogen, ThermoFisher, Waltham, MA, USA) for subsequent experiments prior to treatment and lysates’ generation. In short, the cells were suspended at a density of 10 × 10^6^/mL in PBS and incubated with 2 µM CFDA-SE for 5 min at 37 °C, 5% CO_2_, followed by 1× wash (PBS). Then, cells were incubated in GL261 medium (10 × 10^6^ cells/mL) for another 30 min at 37 °C, 5% CO_2_ and washed again (PBS).

### 4.8. DC Generation and Culturing

Bone marrow-derived DCs were generated, as described [50,51,52]. In short, femurs and tibias of 8–10-week-old C57Bl/J6 mice were removed. The purified intact bones were rinsed in 70% ethanol for 2 min and washed with PBS. Subsequently, the ends were cut off and the bone marrow flushed with PBS using a syringe with a 27G needle and strained through a 50 µm mesh (CellTrics, sysmex, Norderstedt, Germany) into a 50 mL tube. Then, the cell suspension was centrifuged at 500× *g* for 8 min at room temperature (RT). The obtained cells were counted and cultured in T75 flasks. The DC culture medium was RPMI-1640 (Gibco, Thermo Fisher) supplemented with 10% heat-inactivated FCS, 100 U/mL penicillin, 100 µg/mL streptomycin, 2 mM L-glutamine, and 50 µM 2-mercaptoethanol. A total of 200 U/mL murine GM-CSF (mGM-CSF, PeproTech 315-03, PeproTech, Rocky Hill, NY, USA) was added at day 0 and 2 and half of the medium was refreshed at day 4. Cell density was held at 10^6^ cells/mL. At day 7, immature non-adherent and semi-adherent DCs were harvested by gently flushing flasks twice with PBS, and collected and centrifuged at 500× *g* for 5 min at RT. The pellet was resuspended in fresh DC medium and the cells were counted. A part of differentiated cells was stored in 10% DMSO (Sigma-Aldrich)/FCS at −80 °C for subsequent use.

### 4.9. T-Cell Generation and Culturing

To generate spleen-derived T cells, the spleens of 8–10-week-old C57Bl/J6 mice were removed, put into a petri dish with fresh, cold PBS, and cut into smaller pieces using a scalpel. Then, the pieces were shredded/crushed carefully with a 1-mL syringe and strained through a 50 µm mesh (CellTrics, sysmex) into a 15 mL tube filled up with PBS. Cell suspension was centrifuged at 300× *g* for 8 min at 4 °C and supernatant was removed. Erythrocytes were eliminated by incubation in lysis buffer (8.2 mg/mL NH_4_Cl, 1 mg/mL KHCO_3_, 0.037 mg/mL Na-EDTA; pH = 7.3) for 90 s on ice, followed by two washes (PBS, 0.5% BSA, 2 mM EDTA). T-cell isolation was conducted by magnetic cell separation (depletion of the magnetically labeled non-target cells) using the Pan T-cell Isolation Kit II mouse, an LS Column, and a MidiMACS™ Separator (Miltenyi Biotec, Bergisch Gladbach, Germany). Purity was confirmed by the staining of the T-cell marker CD3 and analysis by fluorescence cytometry (FC). Isolated T cells were dissolved in T-cell proliferation medium (RPMI-1640 supplemented with 10% heat-inactivated FCS, 1% HEPES (Lonza), 1% MEM (Gibco, Thermo Fisher), 100 U/mL penicillin, 100 µg/mL streptomycin, and 2 mM L-glutamine) or stored in 10% DMSO/FCS at −80 °C for subsequent use.

### 4.10. DC Pulsing and T-Cell Priming/Repriming

Differentiated DCs (day 7) were pulsed with three different formulations of GL261 lysates (PDT F/T IR; F/T IR; F/T IR PDT) and two formulations of whole GL261 tumor cells (C:IR PDT and C:IR) resulting in five treatment groups per experiment. For loading with GL261 whole cells, 3 × 10^6^ DCs were added at a 1 to 1 ratio and cells were incubated for 1.5 h at 37 °C. Lysate loading was performed in 96-well plates and DCs (3 × 10^6^ cells/250 µL) were incubated with 0.6 mg lysate (corresponding to 3 × 10^6^ lysed GL261 cells) for 1 h at 37 °C. Subsequently after loading, DCs were transferred to a 48-well plate at 1 × 10^6^ DCs per ml in DC medium. To induce maturation, DCs were incubated in the presence of 200 U/mL mGM-CSF and 50 ng/mL LPS (E. coliO111: B4, Sigma-Aldrich) for 24 h at 37 °C, 5% CO_2_. To prime the T cells, loaded mature non-adherent and semi-adherent DCs were harvested by collecting supernatants and by flushing wells with PBS. Half of the non-adherent DCs was stored at −80 °C for reprime. DCs were washed once, resuspended in T-cell medium, and seeded again in a 48-well plate (100,000 DCs/400 µL/well). T cells were added at a 1 to 5 ratio (100,000 DCs: 500,000 T cells). If indicated, reprime was conducted after 72 h of co-culturing (day 11) Therefore, T cells and non-adherent DCs were transferred to a new 48-well plate and the stored mature DCs were resuspended in T-cell medium and added to each well. Additionally, 300 µL/well fresh T-cell medium were added.

The following supplementations were added when indicated: IL4 (recombinant murine IL-4, PeproTech); PD-1-blocking antibody (InVivoMab anti-mouse PD-1, clone: RMP1-14, Hölzel Diagnostika, Köln, Germany)

### 4.11. Cytokine Measurement

To detect the cytokine release by DCs and T cells, supernatants were collected 24 h after the DC pulse, 2–4 days after T-cell prime and 3–5 days after T-cell reprime. Cell debris were removed by centrifugation (10 min, 1300× *g*) and samples stored at −80 °C until measurement. Cytokines were measured using Mouse Flex Sets IL-6, TNF-α or Enhanced Sensitivity Flex Sets for IL4 and IFN-γ (BD Biosciences). Cytometric Bead Array (BD) was conducted according to the manufacturer´s instructions. Samples were measured on a BD Accuri C6 Plus flow cytometer and quantified by FCAP Array v3.0 Software (BD).

### 4.12. T-Cell Proliferation

T-cell proliferation was analyzed by Click-iT EdU assay (Click-iT™ Plus EdU Flow Cytometry Assay Kits; ThermoFisher). T cells were seeded and incubated with T-cell proliferation medium containing a concentration of 3.3 µM EdU. After the respective incubation time, Click-iT EdU assay was performed, corresponding to the manufacturer’s protocol, and analyzed by FC. In some experiments, additional CD4 and CD8 cell-surface antigen staining was conducted.

### 4.13. Cytotoxicity Assays: Calcein Assay

Fluorometric calcein release assay (Calcein Acetotmethylester Viability Dye, eBioscience™, ThermoFisher) was used to analyze the specific cytotoxic activity of T cells against GL261 tumor cells. The GL261 cells were seeded in 96-well plates (10,000 cells/100 µL per well) and allowed to adhere for 24 h. Then, the cells were incubated with 1 µM calcein AM for 25 min at 37 °C, washed once with 200 µL GL261 medium without phenol red (Lonza), and immersed in GL261 medium without phenol red (200 µL/well, containing 0.25 mM probenecid (PromoCell, Heidelberg, Germany) containing activated T cells (four days after repriming). The maximum release control was achieved by using a 0.5% Triton-X 100 (Sigma-Aldrich) solution. Cells were incubated at 37 °C. Four hours after administration of calcein AM, cells were centrifuged at 200× *g* for 5 min, supernatants were transferred to a new 96-well plate (opaque, clear bottom) and fluorescence was measured (Ex/Em = 474/511 nm) on a microplate reader. For measuring calcein AM within the cells, cell brightness was measured on fluorescence microscope (20-fold magnification, Keyence, Neu-Isenburg, Germany) and by FC analysis after carefully detaching cells using PBS. If indicated, the cells were incubated for another 24 h and fluorescence measurement in supernatants was repeated. PD1 block was achieved by incubation of the cells with 25 µg/mL rat anti-mouse InVivoMAb mPD1-AG RMPI1-14 antibody (Biocell, New Delhi, India).

### 4.14. T-Cell Adhesion

The GL261 cells were seeded in 8-well Nunc^®^LabTek^®^ Permanox chamber slides, Simga-Aldrich (8000 cells/well) in GL261 medium. After 24 h, the cells were incubated with T cells (four days after repriming) at a ratio of 5–10:1 T cells to tumor cells for 4 h at 37 °C. Subsequently, the chambers were removed and slides were put into a 50 mL tube filled with serum-free GL261 medium. The cells were fixed with 95% v/v ethanol for 15 min, flushed with PBS, and the slides were covered with gelatin. The adhesion of T cells to GL261 cells was analyzed by counting the proportion of adherent T cells on the GL261 tumor cells using a phase-contrast light microscope (Axiolab, Zeiss Jena, Germany).

### 4.15. Phagocytosis Assay

The frozen, differentiated DCs (day 7) were thawed, resuspended in serum-free DC medium (10 × 10^6^ cells/mL) and transferred to a 96-well plate at 0.5 × 10^6^ cells/50 µL per well. A total of 2 mg/mL of different CFDA-SE labelled GL261 lysate formulations or tumor cells were added to each well and incubated for 120 min at 37 °C, if not otherwise described. Naïve DCs served as the negative control. To analyze specificity, a second plate was treated exactly the same at 4 °C. After incubation time, cells were washed three times with buffer (PBS, 2% BSA) and CD11c staining was conducted. The CFDA-SE and CD11c expression was analyzed by FC.

### 4.16. Fluorescence Microscopy

The GL261 cells were seeded in 8-well Permanox chamber slides (10,000 cells/well) and allowed to adhere. IFNγ (Recombinant Murine IFNγ, PeproTech) at a concentration of 50 ng/mL was added to the appropriate chambers after seeding. After 24 h, the chambers were removed and the cells were fixed with ethanol (96% *v*/*v*) for 15 min. The cells were permeabilized with 0.5% Triton-X 100 (Sigma-Aldrich) in PBS for 5 min and blocked with blocking solution (PBS, 0.25% Triton-X 100) containing 10% goat serum for 30 min at RT. Subsequently, the samples were incubated with the following primary antibodies overnight at 4 °C: PD-L1 (rabbit anti-mouse PD-L1, Abcam, Cambridge, UK) at a dilution of 1:50 (in PBS, 2% goat serum, 0.25% Triton-X 100) or Rabbit IgG Isotype Control Antibody (Abcam) at a solution of 1:170. The slides were washed three times with PBS and the cells were incubated with goat anti-rabbit AlexaFluor568 antibody (Invitrogen, ThermoFisher) at a solution of 1:1000 (in PBS, 2% goat serum, 0.25% Triton-X 100) for 1 h at RT followed by washing with PBS and nuclear staining with 4′, 6-diamidino-2-phenylindole (DAPI, 1:10,000 in distilled water). Slides were covered using Mowiol^®^4-88/DABCO (CarlRoth, Karlsruhe, Germany). Fluorescence microscopy was performed using an Axiolab microscope (Zeiss) and ZEN lite 2.1, Zeiss, software.

### 4.17. Flow Cytometric Analysis (FC)

To analyze the different surface markers, the respective cells (10^5^–10^6^ per batch) were dissolved in 50 µL buffer solution (PBS, 0.5% BSA, 2 mM EDTA) and incubated with 5 µL of primary antibodies for 25 min on ice, followed by washing once with buffer. For the DC staining, one wash step and incubation with 2 µL/10^6^ cells of Mouse BD Fc Block™ (purified rat anti-mouse CD16/CD32, BD Pharmingen, San Diego, CA, USA) for 5 min were conducted prior to the antibody staining. All steps were performed on ice. The following antibodies were used for surface staining: DCs: CD11c (PE hamster anti-mouse CD11c, BD Pharmingen); CD40 (FITC rat anti-mouse CD40, BD Pharmingen); CD86 (PE-Cy7 rat anti-mouse CD86, BD Pharmingen); CD83 (APC rat anti-mouse CD83, BD Pharmingen); NK-cells: CD335 (AlexaFluor647 rat anti-mouse CD335 (NKp46), BD Pharmingen); monocytes: CD14 (APC rat anti-mouse CD14, BD Pharmingen); T cells: CD3 (PerCP-Cy5.5 hamster anti-mouse CD3e, BD Pharmingen); CD4 (PE rat anti-mouse CD4 (RM4-5, BD Pharmingen); CD8 (APC rat anti-mouse CD8a, BD Pharmingen); CD44 (PerCP-Cy 5.5 rat anti-mouse CD44, BD Pharmingen); CD69 (FITC hamster anti-mouse CD69, BD Pharmingen); CD152 (PE hamster anti-mouse CD152, BD Pharmingen). Freshly isolated T cells were stained for PD1 using rat anti-mouse in vivo Mab mPD1-AG RMP1-14 antibody (BioCell, New Delhi, India) and goat anti-rat AlexaFluor488, 1:200, Invitrogen. Flow cytometry was performed using BD Accuri C6 Plus flow cytometer and analyzed using BD Acurri C6 Software/BD C6 Sampler Plus Software. Isotype control stainings served as the negative control.

For PD-L1 surface staining, GL261 tumor cells were cultured in T25 flasks until reaching confluence. Then, 50 ng/mL IFN-γ was added to the respective samples and 24 h later, the cells were harvested using PBS and fixated by incubation with ethanol (96% *v*/*v*) for 10 min, followed by centrifugation at 500× *g* for 5 min. The cells were permeabilized with 0.5% Triton-X 100 in PBS/BSA for 5 min, washed once with PBS/BSA and incubated with the primary antibody PD-L1 (rabbit anti-mouse PD-L1, 1:50, Abcam, Cambridge, UK) or corresponding rabbit IgG isotype control (Abcam) for 25 min at RT. The samples were washed with PBS/BSA and incubated with secondary antibody (goat anti-rabbit Alexa Fluor 488, 1:200, ThermoFisher) for 20 min and washed again before the FC analysis. Freshly isolated T cells were similarly stained for PD-1, using rat anti-mouse in vivo Mab mPD1-AG RMP1-14 antibody (BioCell) and goat anti-rat AlexaFluor488, 1:200 secondary antibody.

### 4.18. Statistics

Statistical significance was assessed by Student’s *t*-test on log data if normal distribution was not fulfilled (*, *p* ≤ 0.05; **, *p* ≤ 0.01, ***, *p* ≤ 0.001). All data are presented as means ± standard error of mean (SEM) if not otherwise written. *n* is defined as the number of independent experiments, if not otherwise described.

## Data Availability

The data presented in this study are available on request from the corresponding author.

## Abbreviations

GBM: glioblastoma multiforme; IR: ionizing radiation; PDT: photodynamic therapy; X-PDT: X-ray-activated PDT; ICD: immunogenic cell death; ROS: reactive oxygen species; LDH: lactate dehydrogenase; THPTS: tetrahydroporphyrin-tetratosylat; BrdU: Bromdesoxyuridin; PD-L1: programmed death-ligand 1; TLR: toll-like receptor; Th: T helper; F/T: freeze/thaw.

## Appendix A

Appendix A data associated with this article. (a) Chemical structure; (b) absorption UV-Vis spectrum of THPTS in acetonitrile (green) and water (black) at room temperature, UV-2101 PC (Shimadzu) UV-Vis spectrometer [32].
